# Whole-Genome Resequencing Reveals Genetic Diversity and Growth Trait-Related Genes in Pinan Cattle

**DOI:** 10.3390/ani14152163

**Published:** 2024-07-25

**Authors:** Dongdong Bo, Yuqing Feng, Yilin Bai, Jing Li, Yuanyuan Wang, Zerui You, Jiameng Shen, Yueyu Bai

**Affiliations:** 1 School of Agricultural Sciences, Zhengzhou University, Zhengzhou 450001, China; bodongd2010@126.com (D.B.); fengyuqing0397@163.com (Y.F.); bylin213@zzu.edu.cn (Y.B.); chnlijing@zzu.edu.cn (J.L.); wyshll2022@163.com (Y.W.); yzr077@163.com (Z.Y.); 13331619207@163.com (J.S.); 2 Key Laboratory of Innovative Utilization of Local Cattle and Sheep Germplasm Resources (Co-Construction by Ministry and Province), Ministry of Agriculture and Rural Affairs, Zhengzhou 450001, China; 3 Henan Animal Health Supervision, Zhengzhou 450046, China

**Keywords:** Pinan cattle, whole-genome resequencing, population genetic structure, selection signature, candidate genes, *GCLC* gene

## Abstract

**Simple Summary:**

Whole-genome resequencing technology is widely applicated in the genetic breeding research of beef cattle. Pinan cattle are a specialized beef cattle population bred in Henan Province for more than 30 years. It has advantages in traits such as a fast growth rate, good reproductive performance, and high slaughter rate. But the genetic variation at the genome level of Pinan cattle has not been thoroughly explored. The whole-genome resequencing of a Pinan cattle population was conducted, combined with genome resequencing data of other cattle breeds, to detect the variations at the whole-genome level. Furthermore, the population genetic diversity and structure were analyzed, and the genes and loci under positive selection affecting important traits of Pinan cattle were identified using a selective sweep strategy. This study contributes to accelerating further breeding of the economic traits such as growth and reproductive performance in Pinan cattle.

**Abstract:**

The breeding of high-quality beef cattle breeds is crucial for the development of animal husbandry, and whole-genome resequencing is widely applicated in the field of molecular breeding. Advantages in growth and reproductive traits exist in Pinan cattle compared with other cattle breeds, but there is limited research on its genomic mechanism. Using whole-genome resequencing, the genetic structure and genomic selection signatures in Pinan cattle were investigated in this study. Phylogenetic, cluster, and admixture analysis results indicated that Pinan cattle have a closer genetic relationship with Kholmogory cattle and China north cattle breeds. Through a selective sweep strategy, 207 and 54 candidate genes related to growth and reproduction and immunity, respectively, were identified in the Pinan cattle population. Given the crucial role of the glutamate–cysteine ligase catalytic (*GCLC*) gene in muscle antioxidative defense, the high frequency of allele T of the *GCLC* c.429 C>T locus in the Pinan cattle population might partially contribute to the advantages of Pinan cattle in growth performance. This study laid the foundation for the genetic improvement in Chinese local beef cattle and provide background for the studies on the growth and development of Pinan cattle.

## 1. Introduction

Henan, as a major agricultural province in China, is also one of the important beef cattle breeding centers in the country. Henan Province produces a large portion of corn, wheat, peanut seedlings, and other crop residues that can be converted into feed every year, providing sufficient feed sources for the development of the beef cattle farming. Furthermore, there are rich beef cattle germplasm resources and nationally recognized beef cattle breeds, such as Xianan cattle, Nanyang cattle [1], and Jiaxian red cattle [2], in Henan Province. These well-known cattle breeds all possess excellent traits including strong adaptability, rapid growth, good meat performance, and high breeding efficiency.

Pinan cattle are a specialized beef cattle group bred in Xinye County, Nanyang City, Henan Province, over more than 30 years. It has the advantages of fast growth, good reproduction performance, high carcass yield, and tight and elastic skin [3]. Under normal feeding conditions, the average daily weight gain of Pinan cattle can be up to 1.62 kg, and the growth rate is 29.2% higher than that of Nanyang cattle [4]. Pinan cattle exhibit an earlier puberty and mating age, and a shorter reproductive interval compared to other Chinese beef cattle breeds [4]. Moreover, Pinan cattle have a high slaughter rate and meat yield, good meat quality, and other excellent traits [5].

Whole-genome resequencing aims at sequencing the genomes of different individuals or populations of a species with a reference genome sequence available. Using this method, researchers are able to analyze genetic variations among individuals and populations; study population genetic structure, genetic equilibrium, and selection [6,7,8]; and uncover the evolutionary mechanisms of the species, environmental adaptation, and molecular-level selection [9]. The identification of candidate genes accelerates the utilization of germplasm resources and the selection breeding of new breeds. With the continuous maturation of sequencing technology and the increasing number of reference genome sequences restored in public databases, whole-genome resequencing has emerged as an effective tool for animal breeding and population evolution research [10].

The identification of genes related to growth, reproduction, and other important traits is crucial to accelerate the breeding process. However, there are limited studies on the genome of Pinan cattle, and the genetic variation at the genome level has not been deeply explored. Therefore, this study aims to investigate the genetic diversity and selection of the Pinan cattle population. In this study, the genome resequencing data of 114 cattle from different geographical regions, including ten Pinan cattle, were used to analyze the genetic diversity and structure of each cattle population. The selective sweep strategy was used to identify genes and loci under positive selection, which might affect the important traits of the Pinan cattle. This study contributes to the establishment of a solid foundation for the genetic mechanism research of important traits in beef cattle, and also provides useful materials for the continuous breeding of Pinan cattle.

## 2. Materials and Methods

### 2.1. Ethics Statement

All experimental work on cattle was approved by the Animal Ethics Committee of Zhengzhou University (Approval ID: ZZUIRB2024-102, approval date: 4 February 2024). Blood was obtained from a beef cattle farm in Xinye County, Henan Province. The specimens were collected by trained personnel and specialized veterinarians following the animal welfare protocols of Zhengzhou University. All methods were performed in accordance with the relevant guidelines and regulations with including a statement in the Declarations Section to this effect.

### 2.2. Sample Collection, DNA Extraction, and Genome Resequencing

Blood samples of Pinan cattle (*n* = 10) were collected from Xinye County, Henan Province. Genomic DNA was extracted from the blood samples with QIAamp DNA Blood Mini Kits (Qiagen, Shanghai, China) in a laboratory, and then the DNA integrity and concentration were checked with 1% agarose gel electrophoresis and Qubit^®^ 3.0 Flurometer (Invitrogen, Shanghai, China), respectively. A DNA library was constructed for each sample (300 bp insert size), and whole-genome resequencing was carried out using DNBSEQ-T7 PE150 at Yingzi Gene Technology (Wuhan, China). The sequencing data have been deposited in the Genome Sequence Archive that is publicly accessible (accession number: CRA016186) at https://ngdc.cncb.ac.cn/gsa (accessed on 1 May 2024).

In addition, after the comprehensive consideration of the representativeness of cattle population geographic distribution, sequencing depth, and data quality, the resequencing data of 104 cattle (Table S1) were acquired from the Sequence Read Archive (SRA) database (https://www.ncbi.nlm.nih.gov/sra, assessed on 20 February 2024). These cattle include Ethiopian breeds (Afar cattle, *n* = 5; Bale cattle, *n* = 10; Bagaria cattle, *n* = 10; Semien cattle, *n* = 10), Russian breed (Kholmogory cattle, *n* = 7), and Chinese breeds (Wenshan cattle, *n* = 7; Wannan cattle, *n* = 5; Leiqiong cattle, *n* = 3; Xiangjiang Brown cattle, *n* = 5; Xiangxi cattle, *n* = 23; Yanbian cattle, *n* = 6; Qaidam cattle, *n* = 3; Anxi cattle, *n* = 3; Zhangmu cattle, *n* = 3; Dianzhong cattle, *n* = 4). In total, the genome resequencing data of 114 cattle were used for the subsequent analysis.

### 2.3. Read Mapping and Single-Nucleotide Variant (SNV) Calling

Using Trimmomatic (v0.39), sequencing adapters and low-quality reads were trimmed with the parameters “SLIDINGWINDOW:5:20 LEADING:5 TRAILING:5 MINLEN:50” [11]. Quality control of filtered data was performed using FastQC (v0.12.1) [12]. All clean reads were aligned to the reference genome (ARS-UCD1.2) using BWA software (v0.7.17) with default parameters of the MEM module [13]. Using SAMtools (v1.16.1), the sequencing depth and coverage were calculated [14]. PCR duplicates were removed with the MarkDuplicates module in the Picard tool (https://broadinstitute.github.io/picard/, assessed on 9 April 2024).

SNV calling was performed by Genome Analysis Toolkit (GATK, v4.4.0.0), including the HaplotypeCaller, CombineGVCFs, GenotypeGVCFs, and VariantFiltration modules [15]. The gvcf files were created using the HaplotypeCaller module and then jointly genotyped to obtain the final vcf file using the CombineGVCFs and GenotypeGVCFs’ modules. The variants were filtered using the VariantFiltration module with the following criteria: (1) depth (DP) < 10; (2) quality by depth (QD) < 2.0; (3) Fisher strand (FS) > 60.0; (4) strand odds ratio (SOR) > 4.0; (5) mapping quality (MQ) < 40.0; (6) mapping quality rank sum test (MQRankSum) < −12.5; (7) read position rank sum test (ReadPosRankSum) < −8.0. The Filtered SNVs were further filtered using VCFtools (v0.1.16) with minor allele frequency (MAF) less than 0.05 [16], followed by the annotation of remaining SNVs using the VEP tool [17].

### 2.4. Population Genetic Structure and Genetic Diversity Analysis

For the principal component analysis (PCA) and admixture analysis, the SNVs were filtered using PLINK (v1.90) with MAF < 0.01, and the LD-based pruning was performed with the ‘--inep-pairwise 50 10 0.1’ option [18]. PCA was performed using GCTA (v1.94.1) [19]. ADMIXTURE (v1.3.0) was used to quantify the genome-wide admixture among cattle breeds and run for each possible group number (*k* = 1 to 8), where *k* is the assumed number of ancestries [20]. The phylogenetic tree was constructed using MEGA (v 11) based on the neighbor-joining (NJ) approach [21].

The allele frequency correlation (*r*^2^) was calculated using PopLDdecay (v 3.42) [22] to evaluate the patterns of linkage disequilibrium (LD) decay in the genomes of three populations. With a window size of 100 kb and a step size of 10 kb, the genome-wide nucleotide diversity (*π*) of cattle populations was estimated using VCFtools (v0.1.16).

### 2.5. Detection of Positive Selection Signatures

Genetic distances among populations were analyzed by estimating *F*_ST_ values using PLINK (v1.90) and VCFtools (v0.1.16) with a window size of 100 kb and a step size of 10 kb. The top 5% windows of *F*_ST(native cattle vs. Pinan cattle)_ and *F*_ST(foreign cattle vs. Pinan cattle)_ were selected as genomic regions under selection. The ratios of *θπ_(_*_native cattle)_/*θπ_(_*_Pinan cattle)_ and *θπ_(_*_foreign cattle)_/*θπ_(_*_Pinan cattle)_ were calculated, and the top 5% windows of *θπ* ratios were considered as genomic regions under selection. The Tajima’s *D* values for each population were calculated separately using VCFtools (v0.1.16) with a window size of 100 kb and a step size of 10 kb. The windows with *D* values less than 0 from the Pinan cattle population were considered as genomic regions under positive selection. Finally, the intersect module of bedtools (v2.17.0) was used to integrate the selection signatures identified by *F*_ST_, *Pi*, and Tajima’s *D* methods [23].

### 2.6. GeneSet Enrichment Analysis

In order to further explore the biological functions of genes under positive selection, enrichment analyses of the gene ontology (GO) and Kyoto Encyclopedia of Genes and Genomes (KEGG) pathway were performed using the Metascape website [24]. The genes under positive selection were used as input. The screening criterion for significantly enriched terms was false discovery rate (*FDR*) < 0.05.

### 2.7. Protein Structure Prediction

The amino acid sequences with and without the mutation were inputted into the SWISS-MODEL website [25] for modeling and the structures of proteins were visualized using PyMOL (v 2.6) [26]. Then, the influence of the mutations on the hydrophobicity of the proteins was analyzed using the ProtScale (https://web.expasy.org/protscale/, accessed on 20 April 2024) [27].

## 3. Results

### 3.1. Sequencing and SNV Calling

Deep resequencing of the 10 Pinan cattle was performed, which yielded 889.70 Gb of sequencing data with an average depth of 32.15× (28.57–38.45×) and an average alignment rate of 99.73%. In addition, 104 cattle’s resequencing data (6485.21 Gb) were downloaded from nucleotide archive databases (Table S1) to better compare the differences between Pinan cattle and other cattle breeds. Ultimately, a sequencing dataset of 7374.91 Gb (Table S2) with an average sequencing depth of 22.23× and an average alignment rate of 99.59% was acquired in this study.

**Figure 1:**
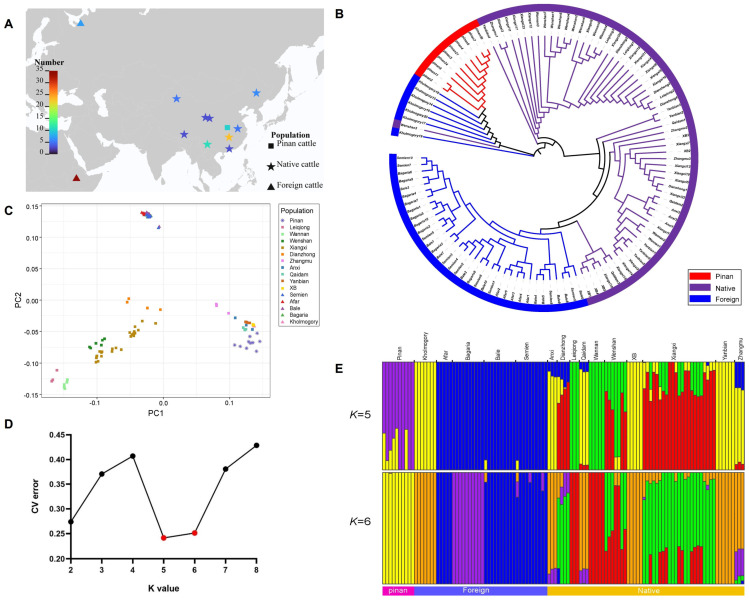
The genetic structure of the cattle populations. (**A**) The geographic distribution of the Pinan cattle population, native cattle population, and foreign cattle population, which are represented by square, pentagon, and triangle dots on the map, respectively. The closer the color is to red, the more individuals there are in that geographic area; the closer the color is to blue, the fewer individuals there are in that geographic area. (**B**) The neighbor-joining (NJ) tree of the 114 individuals. The red, purple, and blue colors in the outermost circle and tree branches represent the Pinan cattle population, the native cattle population, and the foreign cattle population, respectively. (**C**) The PCA plot of the 114 individuals. (**D**) The CV error of the ADMIXTURE analysis. The CV error is minimized when *k* = 5. The red dots represent the CV error when *k* = 5 or 6. (**E**) Model-based clustering of all individuals with *k* = 5 and *k* = 6. Blocks of different colors represent the assumed genetic composition from ancestors.

Then, 114 cattle individuals were classified into three groups (Figure 1A): the Pinan population (Pinan cattle), native cattle population (Wenshan cattle, Wannan cattle, Leiqiong cattle, Xiangjiang Brown cattle, Xiangxi cattle, Yanbian cattle, Qaidam cattle, Anxi cattle, Zhangmu cattle, and Dianzhong cattle), and foreign cattle population (Kholmogory cattle, Afar cattle, Bale cattle, Bagaria cattle, and Semien cattle).

After variant calling and subsequent stringent quality filtering, a total of ~81.43 million SNVs with high quality were finally retained, of which 41,799,790 novel SNVs (not included in the dbSNP database [28]) were identified. The annotation of the genomic locations of these variations revealed that 48.12% of the SNVs are located in intronic regions and 39.19% are located in intergenic regions. A total of 1,711,015 synonymous mutations and 761,210 non-synonymous mutations were identified within the coding regions (Table 1). This SNV dataset provides a new resource for beef cattle biological research and breeding.

### 3.2. Population Genetic Structure Analysis

In order to explore the relationship of genetic structure between the Pinan cattle population and other populations, we constructed a phylogenetic tree and performed a cluster analysis and population structure analysis. The phylogenetic tree showed that the Pinan cattle population clustered with the Kholmogory population, indicating a close genetic relationship; native cattle formed several major branches on the evolutionary tree, with each breed evenly distributed across these branches; four Ethiopian breeds clustered into one branch, suggesting a closer genetic affinity (Figure 1B). PCA was performed using GCTA and the result showed that all individuals were clearly divided into three groups. The Pinan cattle population, native cattle, and Ethiopian cattle from foreign populations were distinctly separated. The Pinan cattle were closer to the northern Chinese breeds (Zhangmu, Anxi, Qaidam, Yanbian, and XB) and significantly distinct from the southern Chinese breeds (Leiqiong, Wannan, Wenshan, Xiangxi, and Dianzhong). Individuals of southern Chinese cattle breeds were relatively dispersed, indicating abundant genomic variation (Figure 1C). A population structure analysis was performed using ADMIXTURE software (v1.3.0). The cross-validation (CV) error is minimized when *k* = 5, so it was speculated that assuming a number of 5 ancestors was more scientifically plausible (Figure 1D,E). The admixture analysis showed that six Pinan individuals exhibited the same ancestry as the Kholmogory population and the cattle population in northern China with a *k* of 5; only one Pinan individual had admixture with the Kholmogory and northern China cattle populations with a *k* of 6.

### 3.3. LD Decay of the Cattle Populations

**Figure 2:**
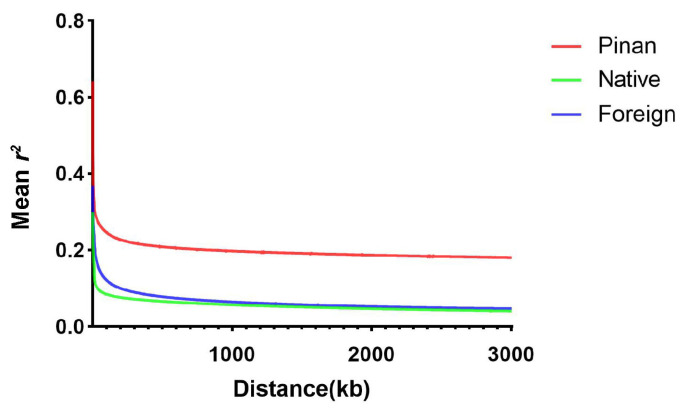
The LD decay of the three populations is shown in the plot. The red, green, and blue curves represent the Pinan population, the native population, and the foreign population, respectively.

LD, in terms of the correlation coefficient (*r*^2^), was calculated for the Pinan population, the native population, and the foreign population. As shown in Figure 2, the LD decay rate of the Pinan cattle population was much lower than that of the native population and the foreign population, suggesting that the Pinan population was subjected to stronger selective pressures at the genomic level.

### 3.4. Positive Selection Signatures in Pinan Cattle Population

**Figure 3:**
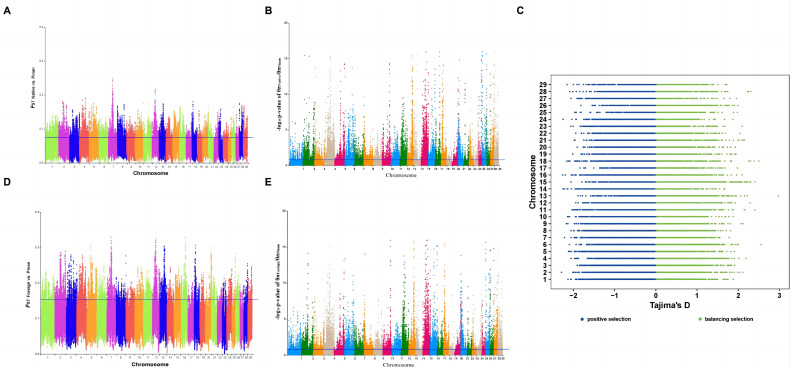
The detection of positive selection signatures in the Pinan cattle population. (**A**,**D**) shows the distribution of paired *F*_ST_ on each chromosome between Pinan cattle and native and foreign cattle populations, respectively; the blue horizontal line represents the 5% *F*_ST_, and the dots located above the line represent the selected regions. (**B**,**E**) are the *θπ* ratio between Pinan cattle and native and foreign cattle populations, respectively. The *p* value was calculated from the Z-normalized *θπ* ratio, and the blue horizontal line represents the *p* value correspondent to 5% of the *θπ* ratio, and the dots located above the line represent the selected regions. (**C**) The result of Tajima’s *D* value in the Pinan cattle population, and the windows with *D* < 0 are considered as positively selected regions.

*F*_ST_ measures genetic differentiation among populations, *π* measures genetic diversity within populations, and the Tajima’s *D* test measures the allele frequency distribution of nucleotide sequence data [29]. Together, they can improve the detection accuracy of selection signatures and provide a more comprehensive view of genetic variation. In this study, we considered genomic regions that simultaneously met the criteria of the top 5% *F*_ST_, top 5% *θπ* ratio, and *D* < 0 (in the Pinan population) as positive selected regions (Figure 3).

**Figure 4:**
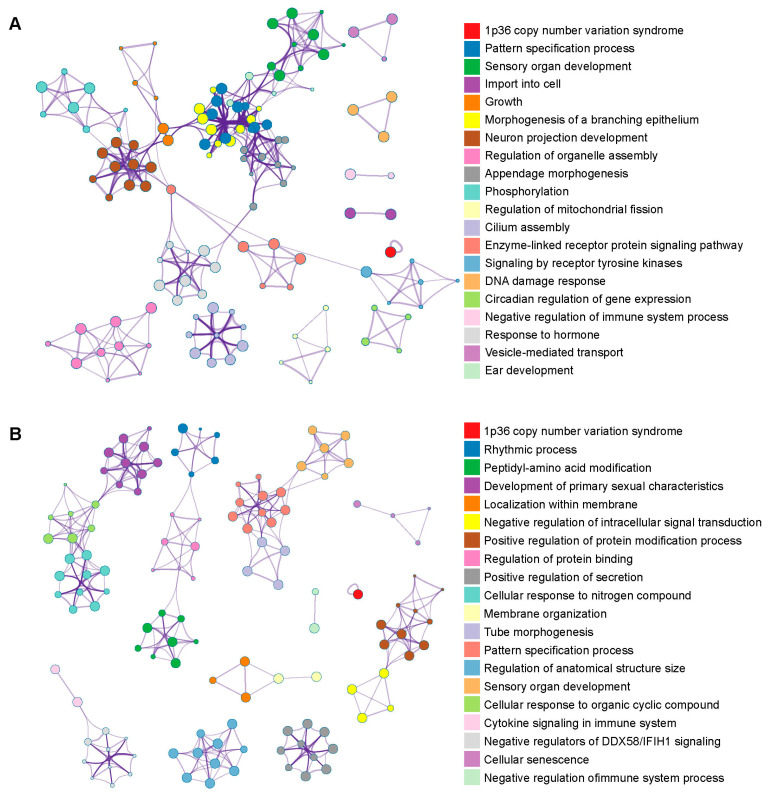
Functional enrichment in the selection signatures. The plot shows the GO and pathway enrichment in the selection signatures of Pinan cattle compared with the native cattle population (**A**) and foreign cattle population (**B**).

In total, 822 and 973 genes in the positive selected regions of the Pinan cattle population compared to native (Figure 4A) and foreign (Figure 4B) populations were identified, respectively. Through functional enrichment, 551 and 723 terms enriched in genes under positive selection were identified compared to native (Figure 4C) and foreign (Figure 4D) populations, respectively. Several terms were associated with growth, reproduction, and immunity. Terms related to growth and reproduction include sensory organ development (GO:0007423), morphogenesis of a branching epithelium (GO:0061138), appendage morphogenesis (GO:0035107), cilium assembly (GO:0060271), response to hormone (GO:0009725), growth (GO:0040007), ear development (GO:0043583), development of primary male sexual characteristics (GO:0045137), and rhythmic process (GO:0048511); terms related to immunity include cytokine signaling in the immune system and the negative regulation of immune response (GO:0002683). A total of 207 and 54 candidate genes related to growth and reproduction and immunity were identified in these terms, respectively (Table S3).

### 3.5. Protein Analysis and Validation of the Positively Selected Locus GCLC c.429C>T in Pinan Cattle Population

**Figure 5:**
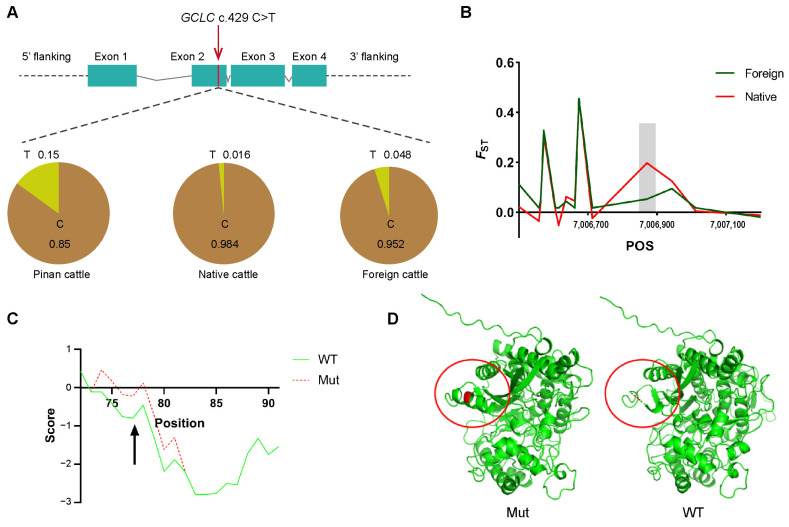
The effect of positive selected locus *GCLC* c.429C>T on the structure and function of GCLC protein. (**A**) The position of *GCLC* c.429C>T in the GCLC gene and the allele frequency in three populations. (**B**) *F*_ST_ values for the *GCLC* c.429C>T locus and nearby loci in the *GCLC* gene. The gray bar represents the nearby region of *GCLC* c.429C>T. (**C**) The green line and red dashed line represent the hydrophobicity profiles of the wild-type (WT) and mutant (Mut) proteins, respectively. Arrows point to the position of the amino acid residue encoded by *GCLC* c.429C>T. (**D**) Protein structures of the mutant (Mut) and wild-type (WT) GCLC proteins. The left illustrates the structure of the mutant GCLC protein, while the right illustrates the structure of the wild-type GCLC protein. The red circles represent the alpha helix altered by *GCLC* c.429C>T.

By the further analysis of the missense SNVs in the genes under positive selection, which are related to growth, reproduction, and immunity, we found that the allele frequency of the *GCLC* c.429C>T site located in exon 2 of the *GCLC* gene significantly differed between the Pinan population and native population, as well as the foreign population (Figure 5A). Additionally, strong selective features were also detected among this site (Figure 5B). Next, we analyzed the impact of *GCLC* c.429C>T on the structure and function of the GCLC protein. *GCLC* c.429C>T increased the hydrophobicity of the encoded amino acid and neighboring residues, impacting the hydrophobicity of the whole GCLC protein (Figure 5C). The change in hydrophobicity may affect the tertiary structure of the protein. Compared to the mutant GCLC protein, the wild-type protein lacks an α-helix (Figure 5D). α-Helices are common protein secondary structures, and their impact on protein structure and function is multifaceted, influencing stability, structural compactness, functional properties, and structural diversity. GCLC is one of the key enzymes in the glutathione synthesis pathway, mainly responsible for catalyzing the reaction between glutamate and cysteine to form the glutathione precursor, γ-glutamylcysteine. Thus, the *GCLC* gene plays a crucial role in regulating the rate of glutathione synthesis within the cell, and its activity and expression level are critical for maintaining cellular oxidative balance. *GCLC* c.429C>T might severely affect the activity of GCLC of protein, such as its ability to bind with specific substrates or cofactors. Given the crucial role of the *GCLC* gene in muscle antioxidant defense, this finding may partly explain the advantage of Pinan cattle in muscle development and production performance compared with other cattle populations.

## 4. Discussion

### 4.1. Population Genetic Analysis

The results of the NJ tree, PCA, and ADMIXTURE analysis indicated that Pinan cattle shared close genetic relationships with Kholmogory cattle and northern China breeds (Zhangmu, Anxi, Qaidam, Yanbian, XB cattle), while being distinct from southern China breeds (Leiqiong, Wannan, Wenshan, Xiangxi, Dianzhong cattle). Huang et al. revealed, based on whole-genome resequencing technology, that most individuals of local cattle breeds from southern and northern China exhibit varying degrees of introgression, but significant differentiation exists between the two populations [30]. Additionally, a study suggested that most northern Chinese cattle breeds are influenced by Bos taurus, while southern Chinese cattle breeds are heavily influenced by Bos indicus [31]. Kholmogory, as one of the native cattle breeds in Russia, is bred by the hybridization between Holstein cattle and local breeds, both of which are Bos taurus. Zhang et al. found that the genetic characteristics of Pinan cattle are significantly influenced by the Bos taurus ancestry from Piedmontese cattle [32]. The conclusions from the above studies are mutually corroborated with the results of the population genetic structure analysis in this study. This might be an important reason why the results of the NJ tree, PCA, and ADMIXTURE analysis all show a close genetic relationship between Pinan cattle, northern China cattle populations, and Kholmogory populations. Both LD decay analyses indicated that Pinan cattle, as a newly bred beef cattle breed in China, have experienced strong selective pressure at the genomic level in recent decades, resulting in lower genetic variability. These findings suggest that the physical distance between variant sites in the Pinan cattle population is close, and they exhibit a high degree of correlation, indicating a tendency for co-inheritance.

### 4.2. Selection Signature Analysis

Massive excellent traits exist in Pinan cattle, such as a fast growth rate, early sexual maturity, high muscle content, and resistance to rough feeding. This implies that candidate genes related to growth, reproduction, and immunity might be key factors in improving the growth performance of Pinan cattle during the breeding process, directly or indirectly regulating their growth rate, muscle content, body size, and overall production performance.

#### 4.2.1. Positively Selected Genes Related to Growth Traits

Bone morphogenetic protein 2 (*BMP2*) encodes a secreted ligand of the transforming growth factor-β (TGF-β) protein superfamily, which plays a key role in developmental processes such as myofibroblast differentiation, bone and cartilage formation, cardiogenesis, and neurogenesis [33]. Tsuji et al. demonstrated that the *BMP2* gene is an endogenous mediator essential for fracture repair, and it plays an important role in the process of murine fracture healing [34]. The *BMP7* gene, as one of the growth factors of the TGF-β superfamily, is widely involved in embryogenesis, hematopoiesis, neurogenesis, and skeletal morphogenesis as well as the regeneration and repair of a variety of other tissues, and Luo et al. demonstrated the necessity of Bone morphogenetic protein 7 (*BMP7*) in the development of mammalian kidneys and eyes by knockout in a murine model [35]. The fibroblast growth factor receptor 2 (*FGFR2*) gene has been shown to play an important role in cellular proliferation, differentiation, migration, and regulation of embryonic development and is required for normal embryonic patterns, lung morphogenesis, and normal bone and skin development [36]. The Gli family zinc finger 3 (*GLI3*) gene is a key transcription factor in the Hedgehog signaling pathway and is essential for normal limb development, nervous system formation, and vertebrate skeletal morphogenesis [37]. The growth differentiation factor 11 (*GDF11*) gene is a member of the TGF-β superfamily, and studies have revealed its importance in regulating the development of the nervous and organ systems, the regeneration of muscle and bone [38], and the health of the cardiovascular system [39]. The Even-skipped homeobox 2 (*EVX2*) gene plays a role in embryonic development, particularly in the formation of the skeleton and the limbs [40]. Homeobox A1 (*HOXA1*), homeobox A2 (*HOXA2*), homeobox A9 (*HOXA9*), homeobox A10 (*HOXA10*), homeobox A11 (*HOXA11*), homeobox A12 (*HOXD12*), and homeobox A13 (*HOXA13*) genes belong to the homologous box (HOX) gene family, which plays a key role in limb formation, the reproductive system, and the formation of other organs during embryonic development [41]. Functional studies of the *HOXA2* gene in different species have revealed its facial, cranial, and ear developmental role [42]. *HOXA13*-knockout mice exhibit phenotypes similar to those of human HFGS, including abnormalities in the development of limbs and reproductive organs, confirming the critical role of *HOXA13* in the development of limbs and the reproductive system. *HOXA11*, as one of the genes essential for female fertility, is involved in the regulation of uterine development [43].

#### 4.2.2. Positively Selected Genes Related to Reproduction Traits

The non-SMC condensin II complex subunit G2 (*NCAPG2*) gene encodes the regulatory subunit of the cohesin II complex, which together with the cohesin I complex plays a role in chromosome assembly and segregation during mitosis [44]. Therefore, the *NCAPG2* gene is important to ensure the correct distribution of genetic information to daughter cells during cell division, affecting cell proliferation, stability, and accurate transmission of genetic material. The Tudor domain containing 7 (*TDRD7*) gene plays an important role in the process of spermatogenesis, development of germ cells, RNA metabolism, and assembly of protein complexes, and Tu et al. revealed its ocular development, retinal function, and reproductive health [45,46]. The estrogen receptor 2 (*ESR2*) gene is an estrogen receptor involved in regulating estrogen signaling and influencing the expression of estrogen-related genes [47]. The GATA binding protein 4 (*GATA4*) gene is a key transcription factor in the development of the heart, intestines, and reproductive organs [48]. The sperm flagellar 1 (*SPEF1*) gene has been associated with the structure and function of the sperm flagellum [49]. The Septin 7 (*SEPTIN7*) gene has been shown to play an important role in the structural integrity and motility of the sperm tail after meiosis [50], and others have shown that the *SEPTIN7* gene affects its skeletal muscle function through muscle regeneration kinetics [51]. The kinesin light chain 3 (*KLC3*) gene plays a role in the development of the sperm tail and normal sperm function [52]. The tubulin tyrosine ligase like 8 (*TTLL8*) gene is capable of controlling flagellar pulsation, directional sperm swim ability, and male fecundity [53]. Proteins encoded by the storkhead box 1 (*STOX1*) gene play a key role in the transcriptional regulation of cells, especially during development. Specific *STOX1* gene variants have been associated with an increased risk of preeclampsia, revealing an important role in the development and function of the placenta [54]. Follicle-stimulating hormone subunit beta (*FSHB*) encodes the follicle-stimulating hormone β-subunit, which binds to the α-subunit to form the follicle-stimulating hormone (FSH) [55]. FSH is an important gonadal-stimulating hormone that regulates the functions of the reproductive system, including follicle maturation and sperm production [56,57].

#### 4.2.3. Positively Selected Genes Related to Immune Traits

Serum protease inhibitor G1 (*SERPING1*) encodes a C1 inhibitor, a key regulator of the complement system and other serum protease pathways primarily involved in the regulation of complement activation, coagulation, and inflammatory processes [58]. The Fps/Fes-related tyrosine kinase (*FER*) gene encodes a non-receptor tyrosine kinase involved in the regulation of cell migration, cell cycle progression, cell adhesion, and signal transduction pathways that may be implicated in cancer development [59]. G protein-coupled receptor 17 (GPR17) is a G protein-coupled receptor implicated in the regulation of glial cell differentiation and response in the central nervous system [60], particularly during repair after brain injury and ischemia. The hemopoietic cell kinase (*HCK*) gene encodes a non-receptor tyrosine kinase belonging to the Src family. *HCK* plays an important role in leukocytes, especially macrophage and neutrophil development and function, and is involved in immune defense, playing an important role in immune defense and inflammatory responses [61]. Interleukin 6 (*IL6*) and Interleukin 10 (*IL10*) genes are important anti-inflammatory and immunomodulatory factors and are produced by a variety of cell types [62,63], including T cells, B cells, and macrophages. Programmed cell death 1 (*PDCD1*), encoding the PD-1 protein, is essential for the regulation of T cell activity and the maintenance of immune tolerance. PD-1, when bound to its ligand, can inhibit T cell activity and prevent an attack on self-tissues [64]. Signal transducer and activator of transcription 2 (*STAT2*) is mainly involved in the type I interferon signaling pathway and plays an important role in antiviral response and immune regulation [65]. The mitochondrial E3 ubiquitin protein ligase 1 (*MUL1*) gene is important in maintaining the healthy state of the cell by regulating mitochondrial mass and function [66]. The NLR family member X1 (*NLRX1*) gene, a member of the NOD-like receptor family, is involved in the regulation of natural immune responses and pro-mitochondrial function, and maintains host immune homeostasis by modulating inflammatory and antiviral responses [67]. The aryl hydrocarbon receptor (*AHR*) gene is a transcription factor that is activated by sensing exogenous and endogenous compounds. It plays a role in a variety of biological processes, including detoxification, cell proliferation and differentiation, immune response regulation, and embryonic development. Upon binding to its ligand, *AHR* is translocated to the nucleus and affects the expression of specific genes [68]. H2.0 like homeobox (*HLX*), which belongs to the homology heterozygous cassette (HOX) family of genes, is a transcription factor that plays a role mainly in embryonic development and hematopoiesis. It is particularly important for the expansion and differentiation of hematopoietic stem cells as well as the development of the immune system [69]. Interferon-stimulated 15 KDa protein (ISG15) is a widely expressed ubiquitin-like protein involved in the antiviral response through ISGylation, a ubiquitination-like modification process. It can interact directly with viral proteins to inhibit viral replication or act by modulating the immune response of the host. ISG15 is also involved in the regulation of cellular signaling, immune responses, and cell survival [70]. Interferon regulatory factor 2 (IRF2) and interferon regulatory factor 3 (IRF3) are members of the family of interferon-regulating factors involved in the regulation of antiviral responses and intracellular signaling [71,72]. TNF receptor superfamily member 4 (*TNFRSF4*) [73] and TNF receptor superfamily member 18 (*TNFRSF18*) are members of the tumor necrosis factor receptor family and are involved in the regulation of T cell activation and survival [74]. GRB2-related adaptor protein 2 (*GRAP2*) is an adaptor protein involved in T cell receptor- and other receptor-mediated signaling [75]. SH2B adaptor protein 3 (*SH2B3*) affects hematopoietic and immune responses by regulating hematopoietic cell signaling [76].

### 4.3. Positively Selected Locus GCLC c.429C>T in Pinan Cattle Population

The positive selection SNV analysis of missense mutations in candidate genes of important traits in the Pinan cattle population showed that the allele frequency of the c.429 C > T locus located in exon 2 of the *GCLC* gene was significantly different between the Pinan cattle population and other populations, and the strong selection pressure of this locus in the Pinan cattle population was also found. Xu et al. identified a novel heterozygous missense mutation, *FGG* c.1168 G > T, and performed expression studies and a functional analysis to explore its effects on fibrinogen synthesis, secretion, and polymerization. The results showed that the novel heterozygous missense mutation *FGG* c.1168 G > T deteriorates fibrinogen synthesis, secretion, and polymerization by altering the secondary structure of the protein and impairing the “A:a” interaction [77]. *GCLC* c.429 C > T increased the hydrophobicity of the encoded amino acids and adjacent residues. In addition, the wild-type protein lacked an α-helix structure compared with the mutant GCLC protein, and this mutation site might seriously affect the biological activity of GCLC. The role of the *GCLC* gene in muscle oxidative stress is mainly reflected in the regulation of glutathione synthesis in muscle tissue, thereby maintaining intracellular oxidative balance, protecting muscle tissue from oxidative damage, and maintaining the structure and function of muscle tissue. Considering the important role of the *GCLC* gene in muscle anti-oxidative defense, this finding may partially explain the superior growth performance of the Pinan cattle population compared to other cattle breeds or populations.

## 5. Conclusions

Herein, we constructed a high-quality genetic variation map using whole-genome resequencing data of 114 cattle, identifying numerous positively selected genes and SNV loci related to growth, reproduction, and immunity in Pinan cattle. Among them, candidate genes associated with growth and development include *BMP2*, *BMP7, FGFR2*, *GLI3*, and *GDF1*; those related to reproduction include *NCAPG2*, *TDRD7*, *ESR2*, and *SPEF1*; and those involved in immunity include *SERPING1*, *CD59*, *HCK*, *IL6*, and *IL10*. The positively selected SNV locus *GCLC* c.429 C>T in the Pinan cattle population may significantly affect the structure of GCLC protein, and the higher frequency of allele T at this locus in the Pinan population may partially contribute to the advantage of Pinan cattle in growth performance compared with other cattle populations. In summary, the genetic diversity and genetic structure of cattle populations, and genomic selection signatures in Pinan cattle, were analyzed in this study, providing a theoretical basis for the future research on the molecular mechanisms and genetic diversity of important traits in Pinan cattle.

## Figures and Tables

**Table 1:** The distribution of SNV variants in the genome region.

Catalog	SNV Numbers
Upstream	4,343,862
Downstream	4,501,793
CDS	2982
Intron	45,812,978
Intergenic	37,307,988
Splicing	198,390
3’ UTR	338,013
5’ UTR	150,159
Synonymous	1,711,015
Non-synonymous	761,210
ts	54,678,068
tv	12,188,775
ts/tv	4.49

## Data Availability

The raw sequence data reported in this paper have been deposited in the Genome Sequence Archive (accession number: CRA016186) in National Genomics Data Center, China National Center for Bioinformation/Beijing Institute of Genomics, Chinese Academy of Sciences, which are publicly accessible at https://ngdc.cncb.ac.cn/gsa (accessed on 1 May 2024).

## Supplementary Materials

The following supporting information can be downloaded at: https://www.mdpi.com/article/10.3390/ani14152163/s1, Table S1: Information of all individuals; Table S2: Sequencing quality and alignment information for all individuals; Table S3: Positive selected genes in Pinan cattle population.
